# Reduced Insulin-Like Growth Factor-I Effects in the Basal Forebrain of Aging Mouse

**DOI:** 10.3389/fnagi.2021.682388

**Published:** 2021-09-01

**Authors:** Jonathan A. Zegarra-Valdivia, Irene Chaves-Coira, Maria Estrella Fernandez de Sevilla, Laura Martinez-Rachadell, Julio Esparza, Ignacio Torres-Aleman, Angel Nuñez

**Affiliations:** ^1^Cajal Institute (CSIC), Madrid, Spain; ^2^CIBERNED, Madrid, Spain; ^3^Universidad Nacional de San Agustín de Arequipa, Arequipa, Peru; ^4^Achucarro Basque Center for Neuroscience, Leioa, Spain; ^5^Department of Anatomy, Histology and Neurosciences, Universidad Autónoma de Madrid, Madrid, Spain

**Keywords:** cholinergic neurons, IGF-I, diagonal band of Broca, cortical activity, aging

## Abstract

It is known that aging is frequently accompanied by a decline in cognition. Furthermore, aging is associated with lower serum IGF-I levels that may contribute to this deterioration. We studied the effect of IGF-I in neurons of the horizontal diagonal band of Broca (HDB) of young (≤6 months old) and old (≥20-month-old) mice to determine if changes in the response of these neurons to IGF-I occur along with aging. Local injection of IGF-I in the HDB nucleus increased their neuronal activity and induced fast oscillatory activity in the electrocorticogram (ECoG). Furthermore, IGF-I facilitated tactile responses in the primary somatosensory cortex elicited by air-puffs delivered in the whiskers. These excitatory effects decreased in old mice. Immunohistochemistry showed that cholinergic HDB neurons express IGF-I receptors and that IGF-I injection increased the expression of c-fos in young, but not in old animals. IGF-I increased the activity of optogenetically-identified cholinergic neurons in young animals, suggesting that most of the IGF-I-induced excitatory effects were mediated by activation of these neurons. Effects of aging were partially ameliorated by chronic IGF-I treatment in old mice. The present findings suggest that reduced IGF-I activity in old animals participates in age-associated changes in cortical activity.

## Introduction

Aging is a physiological process accompanied by a decline in cognitive performance. In humans, aging has been associated with numerous and diverse changes in the EEG and on sleep, such as increased sleep fragmentation, decreased total sleep time, sleep efficiency, and in the frequency bands of the EEG (Landolt et al., 1996; Luca et al., 2015; Mander et al., 2017). Numerous studies have also provided important insights into the global age-dependent alterations in sleep-wake and EEG architecture in mice (Hasan et al., 2012; Panagiotou et al., 2017; McKillop et al., 2018). However, there are notable discrepancies between species concerning the effects of aging. For example, slow-wave sleep (SWS) is decreased in aged humans, whereas it is enhanced in aged mice (Panagiotou et al., 2017; McKillop et al., 2018). Despite these discrepancies, mouse studies have been considered an excellent model to investigate age-dependent physiological changes. Previous evidence suggests that aging may lead to specific changes in cortical activity. Both animal and human studies have shown that aging is associated with alterations in synaptic transmission and structural synaptic changes (Morrison and Baxter, 2012; Petralia et al., 2014) and a consistent loss of hippocampal synaptic connections (Burke and Barnes, 2006; Morrison and Baxter, 2012).

One of the most crucial structures controlling cortical activity is the basal forebrain (BF). Electrophysiological recordings in the BF combined with EEG recordings have indicated that cortical activation depends on BF inputs to the cortex (Metherate et al., 1992; Nuñez, 1996; Duque et al., 2000; Manns et al., 2000). Most of these effects have been explained by the release of acetylcholine (ACh) in the cortex during wakefulness as well as during rapid eye movement (REM) sleep (Celesia and Jasper, 1966; Jasper and Tessier, 1971; Golmayo et al., 2003; Fournier et al., 2004). Dysregulation of the cholinergic system is implicated in cognitive decline associated with aging and dementia, including Alzheimer’s disease (Gallagher and Colombo, 1995; Grothe et al., 2010). For example, an impaired cholinergic transmission has been associated with age-related disorders in attention and memory storage and retrieval (Decker, 1987; Gallagher and Colombo, 1995; Hasselmo, 1999; Dumas and Newhouse, 2011). Since cholinergic neurons are involved in the facilitation and maintenance of arousal, we hypothesized that their activity might be altered in aged animals, particularly their response to modulatory inputs.

In addition, it has been demonstrated that insulin-like growth factor-I (IGF-I) can be a potent stimulator of neuronal activity, participating in numerous brain processes (see for review Fernandez and Torres-Alemán, 2012; Fernandez et al., 2018). Indeed, IGF-I increases the spontaneous firing rate and response to afferent stimulation in target neurons (Carro et al., 2000; Nuñez et al., 2003; Gazit et al., 2016; Barros-Zulaica et al., 2019). We have recently demonstrated that IGF-I increases orexin neurons’ activity, located in the lateral hypothalamus, involved in controlling the circadian sleep/wake cycle (Zegarra-Valdivia et al., 2020). Reduced serum IGF-I levels have been described during aging in all mammalian species studied (Kenyon, 2001; Trejo et al., 2004; Piriz et al., 2011; Muller et al., 2012; Junnila et al., 2013). Basal and IGF-I-induced activation of the brain IGF-I receptor/Akt/GSK3 pathway were markedly reduced in old mice even though they displayed high levels of brain IGF-I receptors (Muller et al., 2012). The reduction of IGF-I effects could be responsible for the decline of cognitive functions during aging. In the present work, we studied whether IGF-I modulates arousal and cortical activity by activation of BF neurons and whether this modulation decreases during normal aging. Specifically, we studied the effect on neurons located in the horizontal, diagonal band of Broca (HDB) nuclei that provide most of the cholinergic innervation to the sensory, motor, and prefrontal cortices and hippocampus (Duque et al., 2000; Zaborszky et al., 2015; Chaves-Coira et al., 2016, 2018). Our findings showed that IGF-I increased HDB neuronal firing, facilitating cortical activity, but this effect was reduced in old mice.

## Materials and Methods

Experiments were performed on B6Cg-Tg (Chat-COP4_H134R/EYFP, Slc18a3)5Gfng/J mice (The Jackson Laboratory). We used these transgenic mice because they express the light-activated cation channel, channelrhodopsin-2, tagged with a fluorescent protein (ChR2-YFP) under the control of the choline acetyltransferase promoter in cholinergic neurons (ChAT+ identified neurons). Thus, all ChAT+ identified neurons express the ChR2 and could be stimulated with blue-light in optogenetic experiments. C57BL/6J mice (Harlan Laboratories, Spain) of both sexes were also used. Animals were grouped into two age groups (young mice: 3–6 months old) and (old mice: 20–22 months old). All experimental groups were sex-balanced.

Animals were housed under standard colony conditions with food and water supplied ad libitum and under a 12–12 h light-dark cycle. Animal procedures followed European guidelines (2010/63, European Council Directives) and were approved by the local Bioethics Committee (Government of the Community of Madrid; PROEX: 189/16). Efforts were made to minimize animal suffering as well as to reduce the number of animals used.

### Recordings and Tactile Stimulation

Animals were anesthetized with isoflurane (2% induction; 1–1.5% maintenance doses) and placed in a David Kopf stereotaxic apparatus (Tujunga, CA, USA). Body temperature was set at 37°C through a water-heated pad (Gaymar T/Pump, Orchard Park, NY, USA). The sagittal midline of the scalp was sectioned and retracted. A small craniotomy was drilled over the HDB nucleus (coordinates from Bregma: 0.5 mm anterior 0.8 mm lateral, 5.5 mm–6 mm depth from the cortical surface).

Multiunit activity (MUA) was recorded in the HDB nucleus through a tungsten microelectrode (1–2 MΩ, World Precision Instruments, WPI, Sarasota, FL, USA). Electrocorticogram (ECoG) was also recorded in the primary somatosensory (S1) cortex (from Bregma −2 mm posterior, 3 mm lateral, 1 mm depth) through tungsten macroelectrodes (<1 MΩ, WPI). MUA and ECoG were filtered between 0.3 and 3 kHz and 0.3 Hz-100 Hz, respectively, and amplified using a DAM80 preamplifier (WPI). Signals were sampled at 10 or 1 kHz, respectively, through an analog-to-digital converter (Power 1401 data acquisition unit, Cambridge Electronic Design, Cambridge, UK) and fed into a PC for offline analysis with Spike 2 software (Cambridge Electronic Design).

Whisker deflections were evoked by brief air pulses using a pneumatic pressure pump (Picospritzer, Hollis, NH, USA; 1–2 kg/cm^2^, 20 ms duration), delivered through a 1 mm inner diameter polyethylene tube. All whiskers were first trimmed to a length of 5 mm to avoid complex responses due to multiple whiskers’ deflections. The experimental protocol consisted of 60 air pulses delivered to the principal whisker at 0.5 Hz after a control period of 1 min for ECoG basal recording.

### Optogenetic Stimulation and Recording

Optogenetic experiments were performed to identify cholinergic neurons (ChAT+ identified neurons) through blue-light activation, using the transgenic mice (see above). Animals were anesthetized and prepared as indicated above. Optical stimulation of ChR-expressing neurons was achieved with light pulses from a light-emitting diode (LED; Thomas Recording, Germany) delivered through an optrode, composed of a tungsten microelectrode 0.5–0.8 MΩ attached to an optical fiber (core diameter 120 μm; Thomas Recording), stereotaxically positioned in the HDB nucleus. Optogenetic stimulation was applied by long-lasting pulses of 473 nm light (27 stimuli with a duration of 300 ms, each stimulus was repeated every 3 s), with an illumination intensity of <30 mW/mm^2^. Unit recordings were performed through the optrode. They were filtered (0.3–3 kHz) and amplified using a DAM80 preamplifier (WPI). Single-unit activity was sampled at 10 kHz and extracted with the aid of Spike2 software.

### Drugs

IGF-I was locally delivered in the HDB nucleus (10 nM; 0.2 μl; coordinates as above) employing a 1 μl Hamilton syringe. Besides, IGF-I was injected systemically (1 μg/g; i.p.) in other experiments. The muscarinic receptor antagonist atropine (1 mg/Kg in 0.9% NaCl i.p.) was administered 15 min before IGF-I systemic injection to assess whether IGF-I effects were due to activation of muscarinic receptors. Orexin A (Tocris, Spain) was also injected into the HDB nucleus (10 nM; 0.2 μl; coordinates as above).

In a set of experiments, we administrated human IGF-I (hIGF-I) through Alzet osmotic mini-pumps (Model 1004; USA) for chronic administration (Pre-Protech, USA; 50 g/kg/day) in ChAT-ChR2-YFP animals or the vehicle (saline solution). Pumps were implanted subcutaneously between the scapulae, following the manufacturer’s instructions. Treatment lasted 28 days. After that, animals were submitted to electrophysiological recordings as described above.

### Immunohistochemistry

Animals were euthanized with an overdose of pentobarbital (50 mg/kg) and perfused transcardially with saline 0.9% followed by 4% paraformaldehyde in 0.1 N phosphate buffer (PB), pH 7.4. Coronal 50-μm-thick brain sections were cut in a vibratome and collected in PB 0.1 N. Sections were incubated in permeabilization solution (PB 0.1N, Triton X-100, NHS 10%), followed by 24/48 h incubation at 4°C with primary antibody (1:500) in blocking solution (PB 0.1N, Triton X-100, NHS 10%). After washing three times in PB, Alexa-coupled goat/rabbit polyclonal secondary antibodies (1:1,000, Molecular Probes, USA) were used.

Finally, a 1:1,000 dilution in PB of Hoechst 33342 was added for 5 min. Slices were rinsed several times in PB, mounted with gerbatol mounting medium, and allowed to dry. The omission of the primary antibody was used as a control. Confocal analysis was performed in a Leica (Germany) microscope. For double-stained ChAT/c-fos counting, five sections per animal were scored using Matlab and Imaris software. The antibodies used in this study include rabbit polyclonal c-Fos (Abcam, UK, ab190289), rabbit polyclonal IGF-I Receptor-β (Santa Cruz, USA, 713/AC), and rabbit anti-IGF-I receptor β XP (Cell Signaling Technology, USA, 9750), anti-Choline acetyltransferase polyclonal antibody (Merck-Millipore, USA, AB144P), and anti-pAkt (Cell Signaling Technology, USA, 9271).

In some experiments, mice were processed for immunocytochemistry 2 h after ip IGF-I injection to allow the expression of c-fos (see above). IGF-I (1 mg/ml) was first dissolved in acetic acid 1N and then prepared with saline to a final dose of 1 μg/g body weight.

### Cell Image Analysis and Counting

Images (coordinates from Bregma: 0.5 mm anterior 0.8 mm lateral, 5.5 mm–6 mm depth from the cortical surface) were taken from the HDB in a confocal microscope (Leica, Germany). The analysis was carried out using the Imaris 9.4 software (the thickness of the images taken was 20 μm). In the ChAT/c-fos analysis, we used 20× magnification; images were taken from the HDB area. Spots of this area were selected with an estimated XY size of 20 μm to count ChAT+ neurons in the red channel. Then, spots were filtered with the c-fos channel in green to obtain the amount of spots ChAT+/c-fos+.

In the ChAT/IGF-1R analysis, we used 40× magnification to measure IGF-IR+ / ChAT+ cells by the signal intensity (%) after identifying the ChAT neurons in the red IGF-1R in the green channel. First, we create a 3D surface (taking the entire thickness) in the red channel using the “surface mode.” In this way, a threshold was set (it was the same for all pictures) to remove the background, and all ChAT neurons above it were selected. Then, we obtained a total number of neurons in the desired area. From the total number of ChAT neurons obtained, we select only those which coincide with the green channel’s maximum intensity, which is the corresponding color of the IGF-1R secondary antibody. To do that, we add a selection filter called “maximum intensity selection in the channel green.” In this way, we were able to distinguish only those neurons that colocalize with the green channel maximum intensity, using an automatic threshold (corresponding to IGF-1R). Finally, we calculated the relationship between ChAT neurons colocalizing with the maximum intensity of IGF-1R.

### Data Analysis

ECoG segments of 1 min every 5 min were analyzed by Spike 2 software, using the Fast Fourier Transform algorithm to obtain the power spectra. The mean power density was calculated for five different frequency bands that constitute the global ECoG: delta (0.3–4 Hz), theta (4–8 Hz), alpha (8–12 Hz), beta (12–30 Hz), and gamma (>30 Hz) bands. The total power of the five frequency bands was considered 100%, and the percentage of each frequency band was calculated. Gamma frequencies were excluded from the results because their power was negligible in anesthetized mice.

Somatosensory evoked potentials (SEPs) were elicited in the S1 cortex by whisker deflections. The area under the curve of the first negative wave was calculated in each case. A control period of 10 min was recorded, and the mean SEP area was considered 100%. Recordings were also performed every 5 min for 30 min after local IGF-I injection in the HDB nucleus. The mean firing rate of MUA recordings was also calculated before (10 min) and after drug injections (every 5 min for 30 min). ChAT+ neurons were identified and included in this study when blue-light pulses induced an increase in their firing rate of at least 10%.

### Statistics

Statistical analysis was performed using Graph Pad Prism 6 software (San Diego, CA, USA). Depending on the number of independent variables, normally distributed data (Kolmogorov–Smirnov normality test), and the experimental groups compared, we used either Student’s *t*-test or two-way ANOVA followed by Sidak’s multiple comparison test. For non-normally-distributed data, we used the Mann–Whitney U test to compare two groups, Kruskall-Wallis or Friedman test, with Dunn’s multiple comparisons and a *Post Hoc* analysis such as Scheirer–Ray Test, a non-parametric alternative to multi-factorial ANOVA. The sample size for each experiment was chosen based on previous experience and aimed to detect at least a *p* < 0.05 in the different tests applied, considering a reduced use of animals. Results are shown as mean ± standard error (SEM) and *p* values coded as follows: *p < 0.05, **p < 0.01, ***p < 0.001.

## Results

### IGF-I Activates HDB Neurons in Young but Not in Old Mice

**Figure 1:**
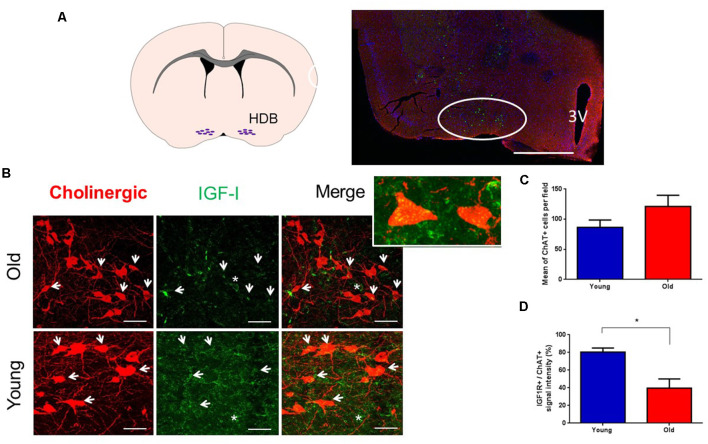
Cholinergic neurons express IGF-I receptors. **(A)** A schematic figure and a representative photomicrograph of the BF showing HDB nucleus (circle) and the ventral putamen. In this case, ChAT+ neurons appear as small green points. **(B)** Photomicrographs of double immunocytochemistry in the HDB nucleus showing staining of cholinergic neurons (red) and IGF-IR (green) in young and old animals. Note the presence of abundant cholinergic fibers in the HDB area and that IGF-IR were expressed in cholinergic (arrows) and non-cholinergic cells (asterisk). Inset shows two ChAT+ cells. **(C)** Mean ChAT+ cell per field in young (86.56 ± 12.11) and old mice (121.2 ± 18.58; *n* = 3 per group; pictures per condition: young = 18, old = 13; *p* = 0.1324, Unpaired *t*-test). **(D)** Measure of IGF-IR+/ChAT+ cells; we observed differences between young (80.3% ± 4.66) and old mice (39.7% ± 10.33; *n* = 3 mice per group, *p < 0.05, Unpaired *t*-test). 3V: Third ventricle. Bar in **(A)**, 500 μm; in **(B)**, 50 μm. Abbreviations: BF, basal forebrain; HDB, horizontal diagonal band of Broca.

Immunohistochemical studies were focused on the HDB area because it projects abundantly to the S1 cortex (e.g., Chaves-Coira et al., 2016, 2018; Figure 1A). Immunohistochemistry of the IGF-I receptor (IGF-IR) showed abundant staining in ChAT + cells in the HDB nucleus of young and old mice. The co-localization of IGF-IR signal to ChAT+ identified neurons strongly suggested that they expressed IGF-IR (Figure 1B). The expression of IGF-IR was also observed in the neuropil and in non-identified cells of HDB. We compared the number of ChAT+ cells per area in young and old mice. There was a slight increase of ChAT+ cells in old animals but differences were not statistically significant (*n* = 3 mice per group, *p* > 0.05; Unpaired *t*-test; Figure 1C). However, we found that the mean expression of IGF-IR in ChAT+ cells of old mice was reduced (*p* = 0.0419; Unpaired *t*-test; Figure 1D), suggesting a reduction of sensitivity to IGF-I in ChAT+ cells of old animals.

**Figure 2:**
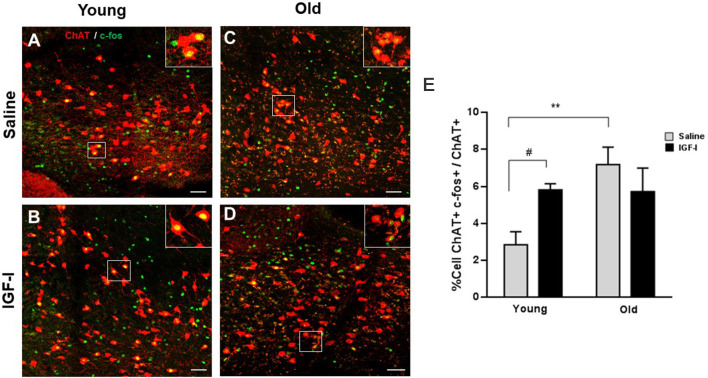
Expression of c-fos in cholinergic neurons of young and old mice. **(A–D)** Representative photomicrographs of HDB area are shown, with a high density of ChAT+ neurons (red) and c-fos expression (green), after saline or IGF-I i.p. injections in young **(A,B)** and old **(C,D)** animals. The bar is 50 μm. **(E)** Plot of the percentage of total ChAT+/c-fos+ cells. While young mice show an increased c-fos expression in ChAT neurons after i.p. IGF-I (1 μg/kg; *n* = 3 mice per group; from 2.85% ± 0.7 to 5.80% ± 0.32; ^#^*p* < 0.05), old mice do not show significant decreased expression (from 7.16% ± 0.94 to 5.17% ± 1.27; *p* = 0.2792). Note that old mice treated with saline showed higher activation of c-fos than young saline (**p < 0.01). Two-Way ANOVA, and Fisher’s LSD comparison test by group.

Then, we determined the expression of the immediate early gene protein c-fos to test if IGF-I activates HDB neurons. We studied double-labeled ChAT+/c-fos+ cells in young and old animal groups after saline or IGF-I i.p. injections (Figures 2A–D). The plot of the percentage of ChAT+/c-fos+ cells showed a significant increase in old-saline over young-saline animals (*p* = 0.0087; Two-Way ANOVA, and Fisher’s LSD comparison test; Figure 2E), suggesting a basal increase in neuronal activity in old animals. Furthermore, IGF-I injection increased the expression of c-fos in young animals (*p* = 0.045; Two-Way ANOVA, and Fisher’s LSD comparison test). However, IGF-I reduced the number of ChAT+/c-fos+ cells in old animals although differences did not reach statistical significance (Figure 2E).

**Figure 3:**
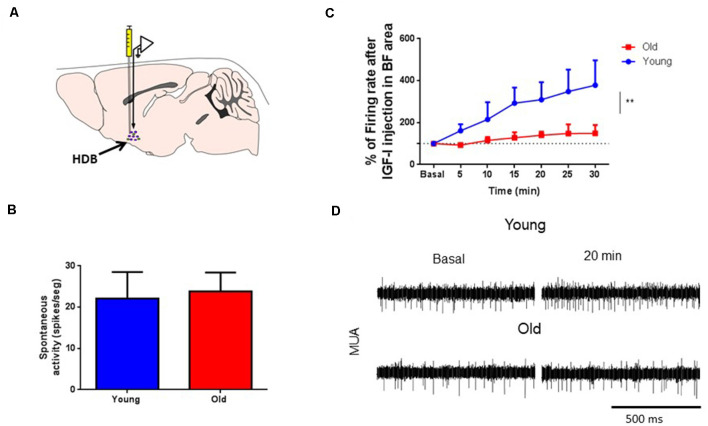
IGF-I increased multiunit (MUA) activity in HDB neurons. **(A)** Schematic diagram of the experimental design. A recording microelectrode was sited in the HDB area (arrow); a cannula to inject IGF-I (10 nM; 0.2 μl) was also placed in the same area. **(B)** Plot of the spontaneous activity in young and old mice. No differences were found in MUA recordings at basal condition (young 22.09 ± 6.41 spikes/s, *n* = 17; old 23.81 ± 4.60 spikes/s, *n* = 9; *p* = 0.2863; sex balanced, Mann–Whitney test). **(C)** Time course of the HDB firing rate after IGF-I local injection. The firing rate of MUA is expressed as a percentage of basal responses at time 0 in both experimental groups. Young but not old mice responded to IGF-I in HDB nucleus (young, *n* = 14; old, *n* = 6; *F*_(1,122)_ = 8,433; **p = 0.0044; Ordinary Two-way ANOVA). **(D)** Representative Multi-unitary recordings (MUA) at basal and 20 min after IGF-I in both young and old mice. The bar represents 500 ms.

The above results suggest a reduced response of HDB neurons to IGF-I in old animals. Consequently, we studied HDB neuronal response to local injection of IGF-I (10 nM; 0.2 μl) through MUA recordings in young and old mice (Figure 3A). We used local injections to avoid a possible reduction of IGF-I entry in old mouse brains through the endothelium. At basal conditions, the spontaneous activity was similar in young and old mice (22.1 ± 6.4 spikes/s and 23.8 ± 4.6 spikes/s, respectively; *p* > 0.05, Unpaired *t*-test; Figure 3B). Local IGF-I injection induced an immediate increase of their firing rate in HDB neurons of young mice (Figure 3C). However, the response to IGF-I was significantly lower in old animals. Five minutes after IGF-I injection, young mice showed an increase of the firing rate of 61.4%, while old animals displayed a reduction of 7.4%. Spontaneous activity continued to increase for at least 30 min (278.1% vs. 48.6% in young and old animals, respectively; young = 14, old = 6; *p* = 0.0044; Ordinary Two-way ANOVA; Figure 3C).

**Figure 4:**
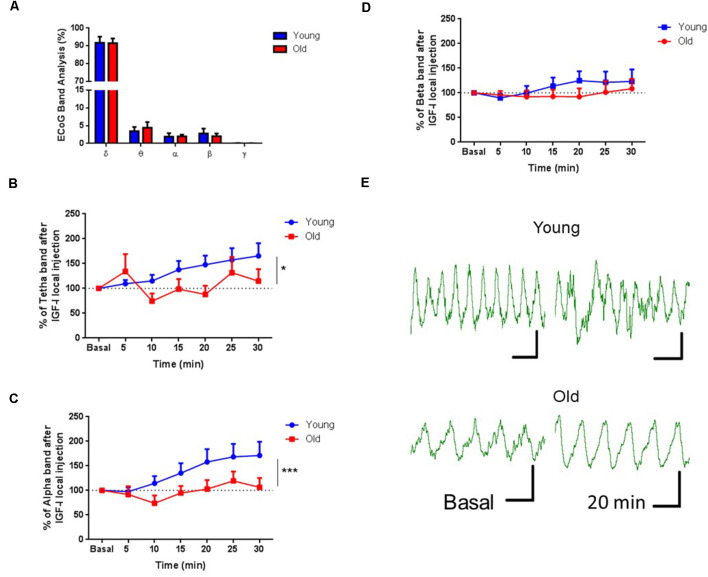
IGF-I injection induced an increase of fast ECoG oscillations. **(A)** Plot of the percentage of the frequency bands calculated from the power spectrum. No differences were observed in the ECoG band analysis of young and old mice previous to IGF-I application (*n* = 9 in each group; *F*_(4,32)_ = 0.04674; *p* = 0.9957, Two-way RM ANOVA). **(B–D)** Time course of each frequency band’s percentage after local injection of IGF-I in the HDB area (10 μM, 0.2 μL). IGF-I induced an increase in the percentage of θ and α oscillations in young but not old mice through 30 min of recording (young = 13, old = 9; *F*_(1,138)_ = 6,436; *p = 0.0123 and *F*_(1,138)_ = 11,85; ***p = 0.0008, respectively; Ordinary Two-way ANOVA. The beta band did not show any difference between groups (young = 13, old = 9; *F*_(1,145)_ = 1.833; *p* = 0.1779; Ordinary Two-way ANOVA). **(E)** Representative EEG recording at basal and 20 min after IGF-I in both young and old mice. The horizontal bar represents 1 s and the vertical bar represents 20 μV.

Since cortical projections of HDB neurons induce cortical activation (Duque et al., 2000; Zaborszky et al., 2015; Chaves-Coira et al., 2018), we tested if the injection of IGF-I in HDB could induce changes in the ECoG recorded in the S1 area of young and old anesthetized mice. We studied the IGF-I effect on the ECoG analyzing its power spectrum and the proportion of the frequency bands. In basal conditions, delta waves were the predominant frequency band due to anesthesia. In these conditions, there were no differences between young and old mice (*n* = 9 per group; *F*_(4,32)_ = 0.04674; *p* = 0.9957, Two-way RM ANOVA; Figure 4A). However, local IGF-I injection in the HDB nucleus (10 nM; 0.2 μl) increased fast ECoG activities in young but not in old mice (Figures 3B–D). θ waves gradually increased their power after IGF-I injection in young animals while old animals only showed a rapid increase at 5 min after injection and returned to basal values, showing a statistically significant difference throughout the 30 min of recording (young = 13, old = 9; *F*_(1,138)_ = 6,436; *p* = 0.0123; Ordinary Two-way ANOVA; Figure 4B). Similarly, α waves increased their power after IGF-I application in young but not in old animals (young = 13, old = 9; *F*_(1,138)_ = 11,85; ***p = 0.0008; Ordinary Two-way ANOVA; Figure 4C). β waves also slightly increased after IGF-I injection in young animals, but differences in this frequency band were small, probably due to anesthesia, and did not reach statistical significance (young = 13, old = 8; *F*_(1,145)_ = 1,833; *p* = 0.1779; Ordinary Two-way ANOVA; Figures 4D,E).

### Tactile Response Facilitation by IGF-I

**Figure 5:**
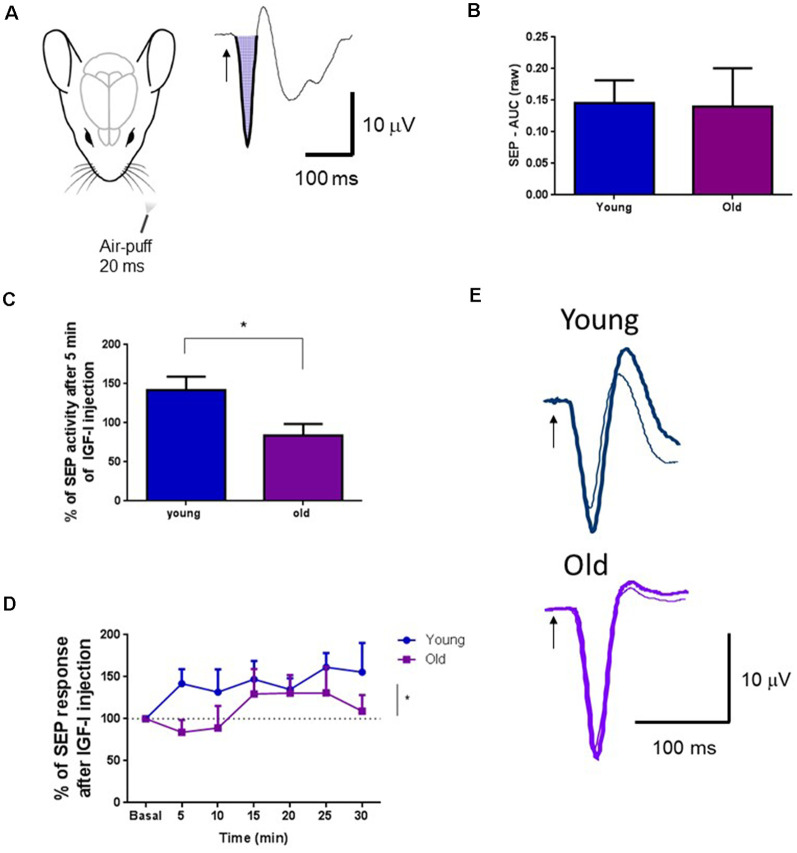
IGF-I facilitated tactile S1 cortical responses. **(A)** Representative diagram of mouse head and air-puff stimulation of whiskers (20 ms duration; left). A representative somatosensory evoked potential (SEP) in the S1 cortex and the calculated area under the curve is shown on the right. **(B)** Plot of the mean area under the curve for young and old mice in basal conditions. There were no differences between young and old mice (young = 14; 0.1520 ± 0.0379 mV^2^; old = 13; 0.1402 ± 0.060 mV^2^; *p* = 0.8682, Unpaired *t*-test). The mean area in basal condition was considered as 100%. **(C)** The SEP area increased in young mice after local injection of IGF-I in the HDB nucleus (10 nM; 0.2 μl) at 5 min after injection while the SEP area was not affected in old mice. Differences between young and old animals were statistically significant (young = 15; 141.9 ± 17.06 μV^2^; old = 13; 83.92 ± 14.71 μV^2^; *p = 0.0176, Unpaired *t*-test). **(D)** Time course of the changes induced by IGF-I local injection in the SEP area. Through time, the IGF-I facilitation was significantly different between groups (young = 15, old = 13; *F*_(1,177)_ = 5.886, *p* = 0.0163; Ordinary Two-way ANOVA). **(E)** Representative SEP in young and old mice, at basal and 5 min after IGF-I injection. Thin traces, basal condition; thick traces, 5 min after IGF-I.

Different studies have demonstrated that BF neurons facilitate cortical sensory responses by activating muscarinic receptors (Golmayo et al., 2003; Fournier et al., 2004; Chaves-Coira et al., 2016, 2018). Here, we want to test if local IGF-I injection in the HDB nucleus also facilitates cortical tactile responses. We analyzed the SEP induced in the S1 cortex by whisker deflections. The SEP area was calculated before and after local injection of IGF-I (10 nM; 0.2 μl) in the HDB nucleus (Figure 5A). We did not find differences in the SEP area between young and old mice at basal condition (young = 14; 0.1520 ± 0.0379 mV^2^; old = 13; 0.1402 ± 0.060 mV^2^; *p* = 0.8682, Unpaired *t*-test; Figure 5B). However, IGF-I injection in the HDB nucleus induced fast facilitation of tactile S1 responses in young mice that increased their response up to 41% over basal response at 5 min after IGF-injection, while IGF-I did not modify tactile responses in old mice at that time (young = 15, old = 13; *p* = 0.0176, Unpaired *t-test*; Figures 5C,E). The time course of tactile responses showed a rapid increase in young mice. Nevertheless, tactile responses slowly increased in old mice, reaching a percentage similar to young animals at 15–25 min after IGF-I application (young = 15, old = 13; *F*_(1,177)_ = 5.886, *p* = 0.0163; Ordinary Two-way ANOVA; Figure 5D).

### Response of Optogenetically Identified Cholinergic Cells to IGF-I

**Figure 6:**
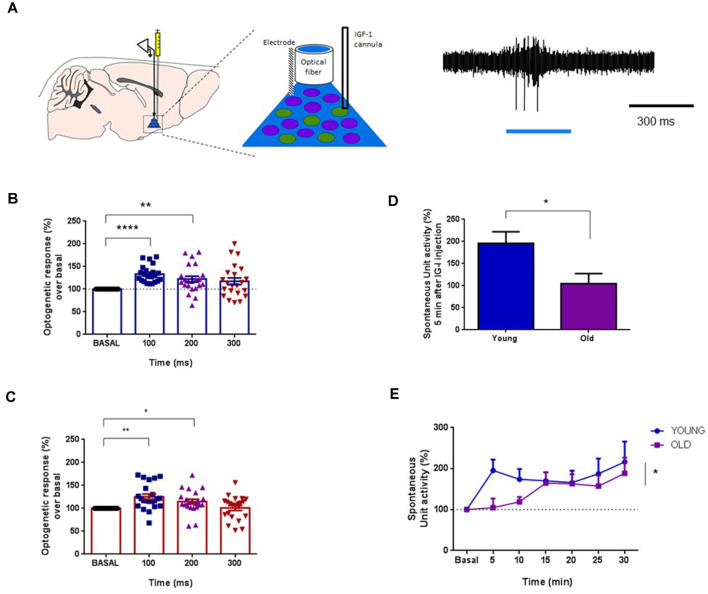
IGF-I increased the activity of optogenetically identified ChAT+ cells. **(A)** Left panel: diagram of the location of the optrode and the cannula in the HDB area. Right panel: optogenetic identification using blue-light pulse (blue bar). Light pulse evoked a spike discharge. **(B)** Plot of the percentage of spike firing with respect to basal activity evoked by the blue-light pulse in young mice (ChAT-ChR2-YFP mice; <6 months old). The firing rate was measured at 100 ms intervals. Neurons were identified as ChAT+ cells when their firing rate increased more than 10% with respect to basal activity in the 0–100 ms time interval. ChAT+ cells increased their firing rate to 133.3 ± 4.01% at 0–100 ms interval, or 122.2 ± 6.55% at 100–200 ms time intervals after the light-pulse onset (*F*_(2.040,42.85)_ = 9.216; *n* = 26 neurons; One-way Repeated Measure ANOVA; at basal vs. 0–100 ms: ****p < 0.0001, or 100–200 ms: **p = 0.0078, Dunnett’s Multiple Comparison Test). **(C)** The same optogenetic identification of ChAT+ cells in old mice (ChAT-ChR2-YFP animals ≥20 months old) mice. The firing rate increased by light-pulses (*F*_(1.908,40.06)_ = 6.702, *p = 0.0035, *n* = 24; One-way Repeated Measure ANOVA; **p < 0.0010 at 0–100 ms vs. basal activity, *p < 0.0277 at 100–200 ms; *n* = 24 neurons; Dunnett’s Multiple Comparison test). **(D)** IGF-I administration induced a 95.7% increase of the firing rate at 5 min in young animals while 4.5% of increase was observed in old animals (*n* = 13 neurons per group; young = 19.57 ± 2.63 spikes/s; old = 10.45 ± 2.28 spikes/s; *p* = 0.0151; Unpaired *t*-test). **(E)** The time course of the IGF-I-evoked response of ChAT+ cells displays significant differences between both groups over time (young = 15, old = 13 neurons per group, *p = 0.0163; Ordinary Two-way ANOVA).

It is well known that cholinergic projections from the BF are responsible for the cortical activation and facilitation of sensory responses (see above references). Consequently, we determine the specific effect of IGF-I on ChAT cells using an optogenetic approach. We used mice expressing channelrhodopsin (ChR) specifically in cholinergic neurons (ChAT-ChR2-YFP animals; see “Materials and Methods” section). We used optogenetic stimulation for selective activation of cholinergic neurons in the HDB nucleus through an optrode that performed unit recordings and optical stimulation simultaneously in the same place. Long-lasting blue-light pulses (300 ms duration) were applied, which stimulated a small volume of tissue (about 200 μm in radius; Figure 6A). Unit activity was measured at 100 ms time intervals after the light onset (0–100 ms, 100–200 ms, and 200–300 ms intervals) and compared with the previous 100 ms time interval before light onset (basal condition). Neurons were considered cholinergic (ChAT neurons) when blue-light pulses induced an increase of at least 10% of their unit activity.

Blue-light pulses induced similar responses in ChAT neurons of either young or old mice (Figures 6B,C, respectively). In young animals, blue-light pulses increased 33.3% the firing rate over the basal level at 100 ms after light onset (*p* = 0.0001), and 22.2% at 200 ms over the basal level (*F*_(2.040,42.85)_ = 9.216; *p* < 0.0004; *n* = 26 neurons; One-way Repeated Measure ANOVA; at basal vs. 0–100 ms: *p* < 0.0001, or 100–200 ms: *p* = 0.0078, Dunnett’s Multiple Comparison test; Figure 6B). Old animals also displayed a blue-light response, which increased at 100 ms (25.4%; *p* = 0.0010) and 200 ms (14.9%; *p* = 0.0277) over basal levels (*F*_(1.908,40.06)_ = 6.702, *p* = 0.0035, *n* = 24; One-way Repeated Measure ANOVA; *p* < 0.0010 at 0–100 ms vs. basal activity, *p* < 0.0277 at 100–200 ms; *n* = 24 neurons; Dunnett’s Multiple Comparison test; Figure 6C). The increment of the firing rate evoked by the blue-light pulse returned to basal levels at 300 ms after light onset in both young and old animals.

After neuronal identification, we studied the effect of local application of IGF-I (10 nM; 0.2 μl) in the HDB nucleus on those neurons. The response pattern of ChAT neurons to IGF-I was substantially different in both animal groups. IGF-I induced an immediate 95.7% increase in the firing rate in young animals at 5 min, whereas only a 4.5% increase was seen in old animals (*n* = 13 neurons per group; young = 19.57 ± 2.63 spikes/s; old = 10.45 ± 2.28 spikes/s; *p* = 0.0151; Unpaired *t*-test; Figure 6D). The time course showed a rapid increase of the ChAT firing rate in young animals while the firing rate increased slowly in old animals (*n* = 13 neurons per group; *p* = 0.0163; Ordinary Two-way ANOVA; Figure 6E).

### Muscarinic Blockade Prevents the Effects of IGF-I in ChAT+ Neurons

**Figure 7:**
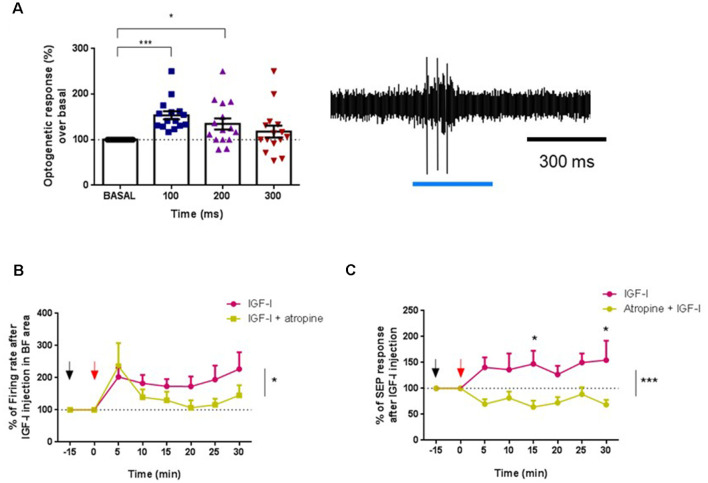
The muscarinic antagonist atropine inhibited IGF-I effects in young animals. **(A)** Optogenetic identification of ChAT+ cells in young (ChAT-ChR2-YFP animals ≤6) before IGF-I administration. Blue-light pulses increased the firing rate activity between 0–100 ms (***p < 0.0001) and 100–200 ms (*p < 0.0345, *n* = 16 neurons per group, *F*_(2,386,33,40)_ = 8,191, One-way Repeated Measure ANOVA; Dunn’s multiple comparison test). Right inset a representative response of a typical ChAT+ cell to a blue-light pulse. **(B)** Fifteen minutes before IGF-I local injection (10 nM; 0.2 μl, red arrow), atropine (1 mg/Kg in 0.9% NaCl, ip, black arrow) or saline (0.9% NaCl) were injected. The firing rate of ChAT+ cells is expressed as a percentage of basal activity at time 0 (IGF-I injection). Young mice administered with saline/IGF-I were compared with young mice injected with atropine/IGF-I. The long-lasting activation evoked by IGF-I was blocked by atropine (saline/IGF-I = 7 neurons, atropine/IGF-I = 9 neurons, *F*_(1,151)_ = 4,878, *p = 0.0287; Two-Way ANOVA). **(C)** SEP facilitation by IGF-I was blocked by atropine i.p. injection. Saline/IGF-I (*n* = 13 neurons) have higher SEP (%) compared with atropine/IGF-I over time (*n* = 9 neurons; *F*_(1,160)_ = 26,38, ***p < 0.0001; Ordinary Two-Way ANOVA), and especially at 15 min (*p = 0.0297 and 30 min *p = 0.216, Sidak’s multiple comparison test).

There is some evidence that IGF-I may interact with muscarinic receptors in various brain systems (Batty et al., 2004; Granja et al., 2019). Thus, we explore if the muscarinic cholinergic receptor antagonist atropine could prevent IGF-I effects in young animals. We firstly identified ChAT cells through optogenetic stimulation, as above. Blue-light pulses increased their firing rate in 16 recorded neurons at 100 ms (53.3%; *p* < 0.001) as well as at 200 ms (34.3%; *p* = 0.0345; Figure 7A). After that, we compared the IGF-I effect on ChAT neurons in control conditions (saline solution i.p.; *n* = 7 neurons) or when atropine was i.p injected 15 min before the local injection of IGF-I in the HDB nucleus (*n* = 9 neurons). In both cases, IGF-I induced a fast increase in the firing rate. However, the long-lasting effect evoked by IGF-I was blocked (saline/IGF-I = 7 neurons, atropine/IGF-I = 9 neurons, *F*_(1,151)_ = 4.878, *p* = 0.0287; Two-Way ANOVA; Figure 7B). This local effect of atropine on the activity of ChAT neurons may be due to the existence of local collaterals within the BF, as has been observed previously (Zaborszky and Duque, 2000; Zaborszky, 2002). Thus, atropine may affect ChAT neurons, reducing spontaneous activity in the HDB as well as the response to IGF-I local application.

In addition, atropine also blocked the SEP facilitation evoked by IGF-I (Figure 7C). IGF-I increased SEP area in control animals (saline-injected animals), while tactile facilitation was blocked in atropine-injected animals (saline/IGF-*I* = 13 animals, atropine/IGF-*I* = 9 animals; *F*_(1,160)_ = 26.38, *p* < 0.0001; Ordinary Two-Way ANOVA).

### Chronic IGF-I Injection Partially Restores Neuronal Responses in Old Mice

**Figure 8:**
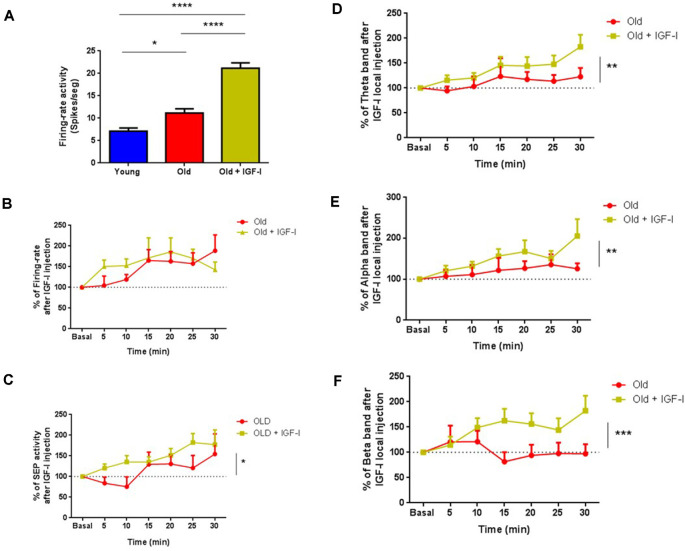
Chronic treatment with hIGF-I recovers cholinergic functions in old mice. **(A)** The firing rate of ChAT+ neurons in old mice (11.19 ± 0.8989, *p = 0.0119; *n* = 10 neurons) and in hIGF-I treated mice (21.17 ± 1.198; ***p < 0.0001; *n* = 16 neurons) were greater than in young animals (7.116 ± 0.7164; *n* = 14 neurons; One-Way ANOVA, ***p < 0.0001, Holm–Sidak’s multiple comparisons test). **(B)** Identified ChAT+ cells shows similar firing rate in old (*n* = 13 neurons) and in old treated animals after IGF-I local injection (*n* = 15 neurons, *F*_(1,182)_ = 0.6,303; *p* = 0.4,283; Ordinary Two-Way ANOVA). **(C)** SEP facilitation was faster in old treated mice compare to old animals over 30 min recordings (old = 13 neurons, old treated = 15 neurons, *F*_(1,180)_ = 5.868, *p = 0.0164, Ordinary Two-Way ANOVA). **(D–F)** ECoG frequency band analysis showed differences between both groups (old = 13 animals, old treated = 15 animals) in θ band (*F*_(1,180)_ = 8.075; **p = 0.0050), α band (*F*_(1,166)_ = 6.790; **p = 0.001) and β band (*F*_(1,182)_ = 14.08; ***p < 0,0002; Ordinary Two-Way ANOVA) over 30 min recordings. ****p < 0.0001.

The above findings showed a reduction of IGF-I effects in old animals. To study if a chronic IGF-I application may restore these effects, we administered IGF-I or saline solution for 28 days through Alzet^®^ mini-pumps in old animals. We compared the spontaneous activity of ChAT+ cells in young and old mice and with ChAT+ cells recorded in IGF-I-treated old mice. Although no differences were observed between the spontaneous firing rate of young and old mice according to the MUA recordings (see Figure 3B), old mice had a higher firing rate (11.19 ± 0.89 spikes/s *n* = 10) than young mice when ChAT+ neurons were specifically recorded (7.12 ± 0.71 spikes/s; *n* = 14 neurons *p* = 0.0119; Figure 8A). Furthermore, ChAT+ neurons in IGF-I-treated old animals showed a greater spontaneous firing rate (21.17 ± 1.19 spikes/s; *n* = 16 neurons) than old untreated (*p* = 0.0001) or young mice (*p* = 0.0001; One-Way ANOVA, *p* < 0.0001, Holm–Sidak’s multiple comparisons test).

Local IGF-I injection in the HDB area (10 nM; 0.2 μl) increased the firing rate in both untreated and IGF-I-treated-old mice. The effect of IGF-I was faster in treated animals; however, differences did not reach statistical significance (*n* = 15 neurons, *F*_(1,182)_ = 0.6303; *p* = 0.4283; Ordinary Two-Way ANOVA; Figure 8B). The IGF-I-facilitatory effect on tactile responses was also faster in IGF-I-treated old mice than in old untreated animals (old = 13 neurons, old treated = 15 neurons, *F*_(1,180)_ = 5.868, *p* = 0.0164, Ordinary Two-Way ANOVA; Figure 8C). In addition, IGF-I-treated old mice show greater fast oscillatory ECoG than untreated old mice after local IGF-I injection in the HDB nucleus (Figures 8D–F). The θ, α, and β frequency bands increased; power of θ band (old = 13, old treated = 15; *F*_(1,180)_ = 8.075; *p* = 0.005; Figure 8D), α band (old = 13, old treated = 15; *F*_(1,166)_ = 6,790; *p* = 0.001; Figure 8E), and β band (young = 15, old = 13; *F*_(1,182)_ = 14.08; *p* < 0.0002, Ordinary Two-Way ANOVA; Figure 8F) over 30 min recording. In conclusion, chronic IGF-I treatment improves basal cholinergic activity and overall cortical activity in old mice.

### Orexin Stimulates Cholinergic Neurons in Young and Old Mice

Finally, we wanted to know whether reduced IGF-I action on ChAT neurons of old animals is a general phenomenon. We tested the effect of orexin in the HDB nucleus because orexin neurons contribute to ECoG activation in the awake state (Sakurai and Sasaki, 1998; de Lecea et al., 1998; Zegarra-Valdivia et al., 2020). Orexinergic receptors are expressed in the BF region and can activate cholinergic and non-cholinergic neurons (Marcus et al., 2001; Arrigoni et al., 2010; Villano et al., 2017). In agreement with that, we observed orexin-A receptors in ChAT+ neurons (Supplementary Figure 1A) and non-identified neurons of the HDB nucleus. Recordings in ChAT neurons of the HDB nucleus of young and old animals (Supplementary Figure 1B) showed an increase in the firing rate in both animal groups after local orexin-A injection (10 nM; 0.2 μl), indicating that orexin responses were not affected by aging (young = 12, old = 8, *p* = 0.6680, Supplementary Figure 1C).

## Discussion

It is well known that healthy aging is frequently accompanied by a decline in cortical activity and with impairment in cognitive information processing. Furthermore, aging is associated with a reduction in the GH-IGF-I axis activity, resulting in lower serum IGF-I levels (Breese et al., 1991) and impaired brain IGF-I activity (Muller et al., 2012). The present study shows that cholinergic-identified and non-identified neurons in the HDB nucleus showed a decreased response to IGF-I in old mice that provoked a reduction of cortical activation and the response to tactile stimuli. We have focused our experiments on the HDB nucleus of the BF because it has been demonstrated that these neurons project to several sensory cortical areas and the prefrontal cortex, controlling their activity (Zaborszky et al., 2015; Chaves-Coira et al., 2016, 2018). Specifically, optogenetic stimulation of cholinergic neurons located in the HDB area facilitated whisker responses in the S1 cortex through activation of muscarinic receptors (Chaves-Coira et al., 2018). The reduction of cholinergic responses with aging effects was partially ameliorated after chronic IGF-I treatment in old mice. Consequently, the present findings suggest that reduced IGF-I responses in the HDB nucleus may explain the cognitive decline observed in old subjects.

IGF-I exerts many actions in the brain, including protection against injury, modulation of neuronal excitability, angiogenesis, or neurogenesis (see for review Fernandez and Torres-Alemán, 2012; Fernandez et al., 2018). IGF-I enhances neuronal activity in many brain areas (Carro et al., 2000; Gonzalez de la Vega et al., 2001; Kelsch et al., 2001; Nuñez et al., 2003; Barros-Zulaica et al., 2019; Zegarra-Valdivia et al., 2020). IGF-I has also been implicated in brain neurotransmitter release regulation, such as ACh neurotransmission. Nevertheless, contradictory results have been observed in *in vitro* experiments. IGF-I decreased ACh release in the hippocampus (Araujo et al., 1989; Seto et al., 2002), while other authors showed an increase of the potassium-induced release of ACh in rat cortical slices by IGF-I (Nilsson et al., 1988). The present results *in vivo* showed that IGF-I application increased the activity of optogenetically identified cholinergic HDB neurons and induced an increase of cFos expression in them. In addition, IGF-I application in the HDB induced fast (>4 Hz) ECoG oscillations and increased tactile responses in the S1 cortex, effects that are mainly due to ACh release in this brain area (Celesia and Jasper, 1966; Jasper and Tessier, 1971; Buzsaki et al., 1988; Golmayo et al., 2003; Fournier et al., 2004). These effects were further supported by the presence of IGF-IR in cholinergic and non-cholinergic HDB neurons. In agreement with that, IGF-I’s facilitatory effect on cortical activity was blocked by systemic injection of the muscarinic receptor antagonist atropine. Note that the IGF-I-evoked increase of cholinergic-identified neurons was also blocked by systemic injection of atropine. This effect may be due to the existence of extensive local collaterals of cholinergic neurons within the BF (Zaborszky and Duque, 2000; Zaborszky, 2002) that may boost the IGF-I local action and thus, blocked by atropine. Thus, atropine may affect local cholinergic action, reducing spontaneous activity in the HDB. Therefore, IGF-I’s effect on cortical activity is, at least in part, due to its action on cholinergic HDB neurons. These results are of great interest because the entry of serum IGF-I into the CNS is more significant during periods of increased neuronal activity or physical activity (Carro et al., 2000; Nishijima et al., 2010). As shown here, systemic IGF-I is known to enhance fast activity in the EEG of mice and non-human primates (Trueba-Sáiz et al., 2013).

Besides, IGF-I increases orexinergic neuronal activity in the lateral hypothalamus, facilitating wakefulness (Zegarra-Valdivia et al., 2020). Therefore, IGF-I facilitates cortical activity in those periods where there is a greater demand for neuronal activity, such as wakefulness, through enhancing cholinergic neurotransmission. The present findings also may explain the reduction of IGF-I effects on cortical activity in an animal model of Alzheimer’s disease (APP/PS1 mouse) in which a reduction of ACh neurotransmission is a hallmark of this disease (Trueba-Sáiz et al., 2013). Therefore, reducing cholinergic neuronal activity by a decrease of IGF-I brain entry/activity can cause the aggravation of different neurodegenerative diseases such as Alzheimer’s, Schizophrenia or Parkinson’s diseases, or aging.

Impairments of cortical activity during aging have been explained by reduced BF neuronal activity, mainly observed in the cholinergic system (Decker, 1987; Gallagher and Colombo, 1995; Hasselmo, 1999; Dumas and Newhouse, 2011). Studies in animals have shown that pharmacological inhibition or neurotoxic lesions of this region cause dramatic impairments in cortical activity, increasing ECoG slow waves and decreasing attention (Buzsaki et al., 1988; Cape and Jones, 1998; Burk and Sarter, 2001; Alenda and Nuñez, 2007; Kaur et al., 2008; Ishii et al., 2017). It has been reported that IGF-I signaling is deteriorated in the brain of aged animals. With increasing age, IGF-I levels decline substantially both centrally (~30% decline) and peripherally (~70% decline) in both humans and rodents (Bando et al., 1991; Sonntag et al., 2005, 2013; Deak and Sonntag, 2012; Ashpole et al., 2015). Basal and IGF-I-induced activation of the brain IGF-I receptor/Akt/GSK3 pathway is markedly reduced in the old brain (Muller et al., 2012).

The present findings show that there is an important change in the BF activity during aging. We found that multiunit activity of HDB area remained equal in young and old animals (Figure 3B); however, when we studied specifically ChAT neurons we found an increase in the spontaneous activity of these neurons recorded in old animals with respect to young animals (Figure 8A). This finding was in agreement with a larger expression of c-fos in ChAT neurons of old animals than in young animals when a saline solution was i.p. injected (Figure 2E). It is well known that there is a reduction of cholinergic functions with aging, but this increase of spontaneous activity of ChAT neurons may not be effective and could be due to a reduction of inhibition from GABAergic cells, as has been described in other neuronal systems (Ling et al., 2005; Ouda and Syka, 2012). In addition, we observed a slight increase of ChAT+ cells in old animals although differences were not statistically significant. It has been indicated previously that the reduction of cholinergic functions is not due to a loss of cholinergic cells (Schliebs and Arendt, 2011). However, the expression of IGF-IR was clearly reduced in old animals. According to these findings, the IGF-I-evoked responses were clearly reduced in old mice. Consequently, the facilitation of tactile responses in the S1 cortex and the activation of ECoG induced by IGF-I was also reduced in old mice. The effect of aging on the IGF-I responses looks specific since responses of HDB neurons to orexin remained intact in old animals (see Supplementary Figure 1).

Therefore, the present results suggest that reduced IGF-I activity may hinder information processing in the cortex, thus explaining the cognitive deficits observed in aging. Mainly, the response of neuronal activity, ECoG, and sensory responses were lower in old animals with respect to young ones. These lower effects may be due to a reduction of the molecular pathways evoked by IGF-I or by a reduction of IGF-IR on ChAT neurons, as has been demonstrated in the present work. Prolonged systemic administration of IGF-I in old mice partially restored IGF-I effects on HDB neurons and cortical activity, as shown here and by other authors (Markowska et al., 1998; Trejo et al., 2007). These beneficial effects may be due to the activation of all available receptors. Certainly, IGF-I administration can prevent loss of cognitive performance in humans (Lupien et al., 2003). Another possibility to increase IGF-I activity is physical exercise. It is known that exercise stimulates brain entrance of circulating IGF-I, which mediates many beneficial exercise actions in the brain (Carro et al., 2000; Trejo et al., 2001). Indeed, our previous findings indicated that physical exercise activates the EEG, increasing the hippocampal theta rhythm and improving memory in healthy mice, suggesting that the exercise-evoked increase of IGF-I may favor cortical activity and memory processes (Miki Stein et al., 2017). In addition, there are also pathological situations in which circulating IGF-signaling is decreased, such as diabetes and Alzheimer’s disease (Torres-Aleman, 2005; Fernandez et al., 2018). Our findings open the possibility for the development of new therapeutic strategies based on increasing circulating IGF-I levels or mimetic drugs of this peptide, potentiating neuronal activity, and specifically the reinforcement of cholinergic activity during pathological conditions or normal aging.

## Data Availability Statement

The raw data supporting the conclusions of this article will be made available by the authors, without undue reservation.

## Ethics Statement

The animal study was reviewed and approved by Animal procedures followed European guidelines (2010/63, European Council Directives) and were approved by the local Bioethics Committee (Government of the Community of Madrid; PROEX: 189/16).

## Author Contributions

JZ-V conducted experiments, prepared figures, results, and wrote part of the manuscript. IC-C, MS, LM-R, and JE conducted immunohistochemistry studies and cell image analysis. IT-A wrote part of the manuscript. AN designed and conducted experiments and wrote part of the manuscript. All authors contributed to the article and approved the submitted version.

## Conflict of Interest

The authors declare that the research was conducted in the absence of any commercial or financial relationships that could be construed as a potential conflict of interest.

## Publisher’s Note

All claims expressed in this article are solely those of the authors and do not necessarily represent those of their affiliated organizations, or those of the publisher, the editors and the reviewers. Any product that may be evaluated in this article, or claim that may be made by its manufacturer, is not guaranteed or endorsed by the publisher.

## Supplementary Material

SUPPLEMENTARY FIGURE 1 **(A)** Diagram of the optrode and the cannula location in the HDB nucleus (upper inset). Lower: Representative photomicrograph of ChAT+ cell (red) and orexin receptor-A. Cholinergic neurons expressed orexin receptor-A. The bar in **(A)** is 50 μm. **(B)** Optogenetic identification of ChAT+ cells in old mice (ChAT-ChR2-YFP animals; ≥18 months old). ChAT+ identified neurons increased their active during light pulse (at 0–100 ms, 120.6 ± 3.916; ****p* < 0.001, and at 100–200 ms, 117.2 ± 6.345; ***p* = 0.0044, *n* = 42 neurons; over basal, 100 ± 0; Friedman test, Dunn’s multiple comparison test. **(C)** Orexin-A injection in HDB (10 nM; 0.2 μl) increased both young and old mice’s firing rate. The firing rate in both experimental groups is expressed as a percentage of basal activity at time 0 (100%), does not find differences between groups (*F*_(1,159)_ = 0.1847; *p* = 0.6680; Ordinary Two-way ANOVA.
