# Supplementary figures and images for: Reduced Insulin-Like Growth Factor-I Effects in the Basal Forebrain of Aging Mouse
Source: Front Aging Neurosci. 2021 Sep 1;13:682388. doi: 10.3389/fnagi.2021.682388 (PMC8442768; doi:10.3389/fnagi.2021.682388)

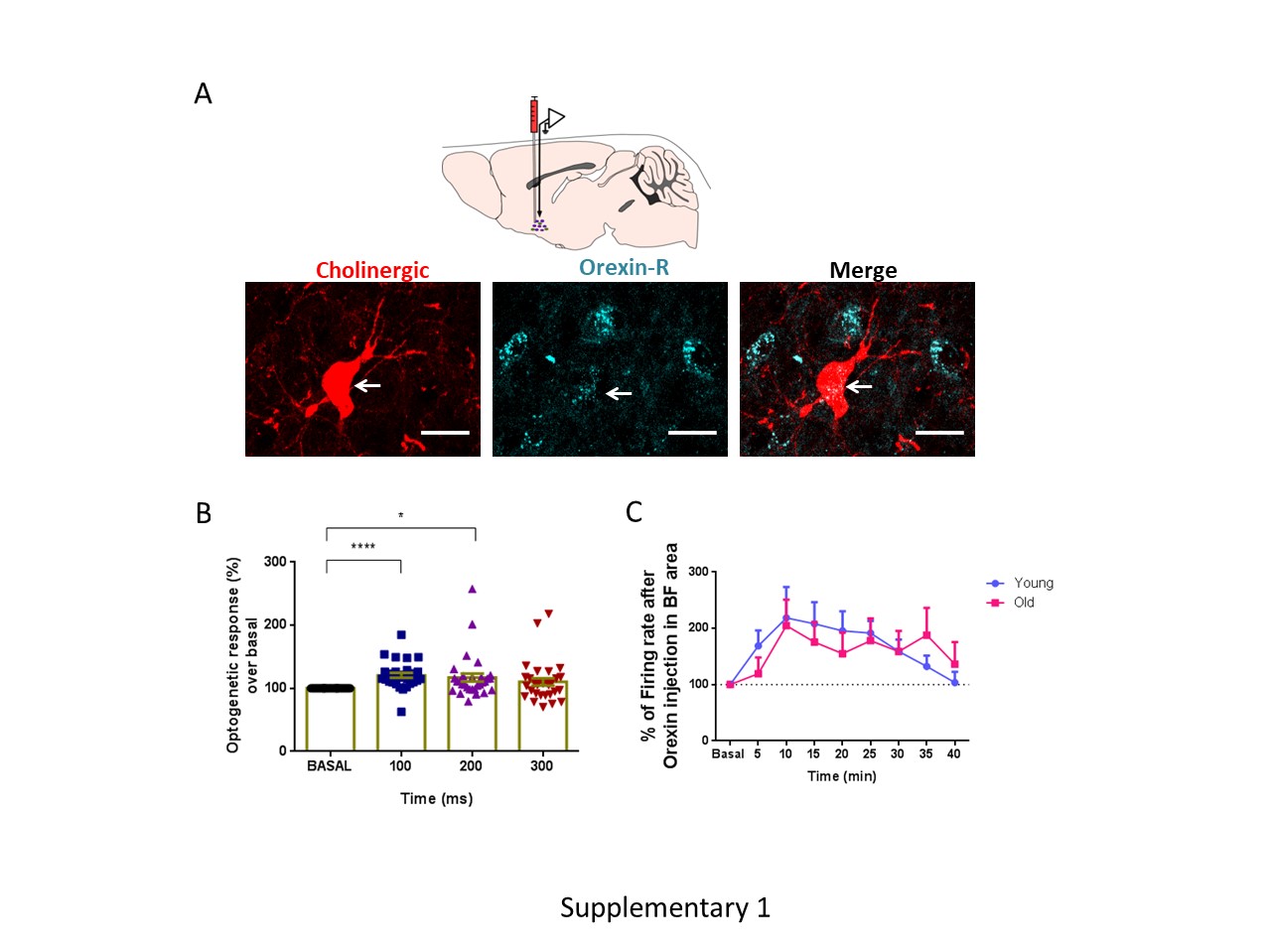

Supplement: SUPPLEMENTARY FIGURE 1 — (A) Diagram of the optrode and the cannula location in the HDB nucleus (upper inset). Lower: Representative photomicrograph of ChAT+ cell (red) and orexin receptor-A. Cholinergic neurons expressed orexin receptor-A. The bar in (A) is 50 μm. (B) Optogenetic identification of ChAT+ cells in old mice (ChAT-ChR2-YFP animals; ≥18 months old). ChAT+ identified neurons increased their active during light pulse (at 0–100 ms, 120.6 ± 3.916; ***p < 0.001, and at 100–200 ms, 117.2 ± 6.345; **p = 0.0044, n = 42 neurons; over basal, 100 ± 0; Friedman test, Dunn’s multiple comparison test. (C) Orexin-A injection in HDB (10 nM; 0.2 μl) increased both young and old mice’s firing rate. The firing rate in both experimental groups is expressed as a percentage of basal activity at time 0 (100%), does not find differences between groups (F(1,159) = 0.1847; p = 0.6680; Ordinary Two-way ANOVA. [file Image_1.jpg]
